# “I’ve always felt like I’m on the outside”: identity and social inclusion among young adults with mental illness and complex needs – a qualitative study

**DOI:** 10.1080/17482631.2024.2433320

**Published:** 2024-12-09

**Authors:** Silje Nord-Baade, Ottar Ness, Michael Rowe, Camilla Bergsve Jensen, Anne Landheim

**Affiliations:** a Innlandet Hospital Trust, Norwegian National Advisory Unit on Concurrent Substance Abuse and Mental Health Disorders, Hamar, Norway; b Department of Health and Nursing Sciences and Section for Mental Health and Rehabilitation, University of Inland Norway, Elverum, Norway; c Department of Education and Lifelong Learning, Faculty of Social and Educational Sciences, Norwegian University of Science and Technology, Trondheim, Norway; d School of Medicine, Department of Psychiatry, Yale Program for Recovery and Community Health, Yale University, New Haven, CT, USA

**Keywords:** Flexible assertive community treatment, identity, mental health, social exclusion, social inclusion, substance use, qualitative study, young adults

## Abstract

**Purpose:**

Addressing social inclusion often involves objective measures and means related to work, education, housing and economy. However, important subjective factors, such as the relationship between identity on social inclusion among young adults with mental illness and complex needs, are understudied. The study objective is to explore how young adults with mental illness and complex needs experience their identity in relation to social inclusion.

**Materials and methods:**

The study adopted a qualitative, explorative, and participatory design. Seven young adults (three males/four females, aged 22–29) were recruited though Flexible Assertive Community Treatment (FACT) Teams, and semi-structured interviews were conducted. Reflexive thematic analysis was employed in the analysis.

**Results:**

The analysis resulted in two main themes. Theme one, “Who they are”, included three subthemes: “A lifelong sense of being someone on the outside”, “Feeling different from others”, and “Someone who is worthless and doesn’t matter”. Theme two, “Who they want to be”, included two subthemes “Wanting to be someone else”, and “The need to redefine oneself”.

**Conclusions:**

This study underlines the importance of working systematically on subjective aspects such as identity, in addition to objective measures, to address social exclusion in a vulnerable and marginalized group. Future directions for research and the development of a more tangible and holistic approach to promote social inclusion are suggested.

## Introduction

According to the United Nations (1, p. 20), social inclusion is defined as improving the terms of participation in society for people who are disadvantaged “[…] through enhanced opportunities, access to resources, voice and respect for rights,” and can be understood as both a process and an outcome. Further, social exclusion is suggested as “a state in which individuals are unable to participate fully in economic, social, political and cultural life, as well as the process leading to and sustaining such a state” (1, p. 18). In addition, there are numerous other definitions of social inclusion with employment, education, housing, neighbourhood, social activities and support most often cited (Filia et al., 2018). Related to social inclusion is the concept of citizenship, defined by its 5 Rs of rights, responsibilities, roles, resources, and relationships that society offers its members through public and social institutions. It also emphasizes the sense of belonging in a society that must be validated by others (Rowe, 2015). The theory of mattering is another approach, addressing wellbeing and social inclusion through people’s sense of feeling like they matter to themselves and others (Prilleltensky & Prilleltensky, 2021; Prilleltensky et al., 2023).

The numerous definitions and approaches have led to debate and critique on the lack of universality in the understanding of the concepts of social inclusion and exclusion. Some argue that it is ambiguous and can be interpreted in various ways (Rawal, 2008). Others call for a shift in focus from exclusion to understanding paths of inclusion (Sekher & Cârciumaru, 2019). Others highlight the need to include both objective and subjective elements to create a comprehensive understanding of social inclusion (Baumgartner & Burns, 2014; Gardner et al., 2019a), arguing that the impact and processes of subjective elements in social inclusion are understudied. Objective measures to promote social inclusion are important, but there is also a need to understand subjective elements.

Social exclusion as both a cause and consequence of mental illness and complex needs is a global challenge and is receiving increased attention in policies, practice, research, and society at large (Goldblatt et al., 2023; United Nations, 2016). The links among, and interactions of, social inclusion, quality of life, physical and mental wellbeing are well documented, as are social exclusion, mortality, morbidity, poverty, poor physical and mental health, and reduced quality of life (Brandt et al., 2022; Goldblatt et al., 2023; United Nations, 2016). Enhancing service providers’ ability to promote health and social inclusion plays an important role in reducing personal costs for the individual, general costs for the society and reducing the pressure on the welfare system (Goldblatt et al., 2023; 2020–2021; NOU, 2023).

While Norway is a well-functioning welfare state compared to many other countries, there are high rates of young adults that are not in school or employed (NEET). People in the NEET category report worse health status than non-NEET peers (Chandler & Santos Lozada, 2021), with social challenges, mental health, and substance use disorders often related to their current situation (Sveinsdottir et al., 2018). Young adults with mental illness and complex needs constitute one of the most marginalized groups of people in our society and internationally (Goldblatt et al., 2023; United Nations, 2016). Research shows that people in these groups often face challenges related to independent living and social reciprocity (Gardner et al., 2019b) and experience stigma through being defined as inadequate by their communities (Semb et al., 2019). These challenges affect all areas in their lives. Younger generations in Norway, and young people internationally, experience lower quality of life and have less faith in the future than older population groups compared to previous years (Goldblatt et al., 2023; Grimstad et al., 2023; Helliwell et al., 2024; Nes et al., 2019). This indicates a societal development that may lead to increased risk of exclusion among already marginalized young adults. Despite increased awareness about the importance of social inclusion, several reports and studies show that Norwegian and international mental health and substance use services pay too little attention to social factors in the treatment of complex problems (Johnson, 2017; Karadzhov, 2023; Norwegian Board of Health Supervision, 2019a, 2019b; The Office of the Auditor General, 2021; Topor et al., 2022; Ventevogel, 2014).

Previous research shows that a negative identity functions as a barrier for social inclusion (Gardner et al., 2019a), as identity defines who we are and how we see ourselves in the community (Birney, 2023). The recovery process of young people with mental illness involves belonging with other young people, in turn affecting their identity formation (Storm et al., 2023). Further, studies find that identity plays a complex and multifaceted role in the social inclusion of young adults with mental illness and complex needs, highlighting the need for further research in this area (Liljeholm & Bejerholm, 2020; Storm et al., 2023). In addition, these people often have extensive experience of negative life events, stigma, and marginalization (Corrigan & Watson, 2002; S. A. Kidd, 2003; S. Kidd & Davidson, 2007). Having such experiences early in life will inevitably create a risk of a continued negative identity development with a sense of one’s lack of self-worth. Consequently, this may lead to social withdrawal and reduced social engagement, despite being offered material means to social inclusion such as housing when homeless (Arnett, 2000; Huxley & Thornicroft, 2003; Thulien et al., 2019). In addition, there is limited research on the relationship between social inclusion and psychological distress among young adults, with identity as one such element of potential distress (Gardner et al., 2019a). Thus, for services working to promote health and social inclusion, it is vital to understand the internal and external processes of influence of social exclusion. The role of identity in the promotion of social inclusion is understudied in this target group and in the context of Nordic countries and the health services.

Studying identity among young adults involves understanding the interplay between individual, social, and societal aspects. At the individual level identity formation is a personal process where young adults explore and define who they are (Birney, 2023). Social aspects (Crocetti et al., 2018, 2023) involve the influence of relationships and social interactions. Societal aspects (Crocetti et al., 2018, 2023) encompass the broader cultural, economic, and political contexts that influence identity. Societal norms, values, and expectations can shape how young adults see themselves and their place in the world. The relationship between these aspects is dynamic and interdependent (Crocetti et al., 2018, 2023).

There are numerous concepts of identity, such as social identity, personal identity, ethnic identity, and gender identity (Birney, 2023). In this study we endorse the narrative theoretical approach that identity is created through a coherent and meaningful life story based on experiences and personal characteristics, shaping our self-concept, self-esteem, and overall wellbeing. The narrative approach emphasizes the dynamics between the individual and societal structures in the creation of these narratives (Arnett, 2000; McAdams, 2001; McLean & Syed, 2015). Employing the narrative approach fits well with the need to explore people’s subjective experiences and perceptions, allowing to tell their stories in their own words and revealing how they make sense of their experiences within their social contexts. As argued by the central theorists, identity is dynamic and develops throughout life (Bruner, 1990; Erikson, 1994; McAdams, 2001; Ricœur, 1992). This approach offers hope that change is possible for a group of people that often may have a negative view of both them and society.

The aim of this qualitative study was to provide practice-oriented knowledge on promotion of social inclusion, providing guidance for practice and further research. To promote social inclusion among young people with mental illness and complex needs, there is a need to gain more in-depth knowledge of individual perspectives of identity, given its impact on how people interact with society and perceive their roles in their communities. The study objective was to explore how young adults with mental illness and complex needs experience their identity in relation to social inclusion.

## Materials and methods

### Design

The study has a qualitative, exploratory, and co-production design influenced by social constructionism (Gergen, 2015, 2022). The social constructionist approach views knowledge as co-created. It enables us to explore how our experiences shape our understanding of the world by combining the factors and dynamics of the personal and social context. This approach is consistent with a narrative approach to identity. A co-production design was chosen to secure the relevance and validity of the study, acknowledging the valuable combination of both lived experience and academic knowledge.

Following Beresford (Beresford, 2013), co-production occurred on both consultative and participatory levels. In the planning phase, the relevance of the study was discussed with different experts and people with lived experience, among these two young adults with extensive lived experience of mental health problems who are members of a national advisory board on the implementation of Youth FACT in Norway. These discussions, which included experts’ comments that they had never before been asked about their possible needs and wishes for social inclusion, confirmed the need for this study. Further, a peer researcher (second author) has participated in the research process, from planning to the dissemination of results.

### Research context

The participants were recruited through receiving services from FACT teams (Flexible Assertive Community Treatment). A FACT team is a multidisciplinary service model that provides integrated care for people with severe mental health and/or substance use disorders (van Veldhuizen, 2007). Implementing FACT teams represents a positive development in Norwegian services that struggle with providing integrated care for people with severe mental health and substance use problems (Landheim & Odden, 2020; Nord-Baade et al., 2022). In Norway, the teams are a collaboration between specialist health care and municipal services and draw on expertise from both levels in addition to peer support. Social inclusion is an integral part of the model, with provision of services in the local community and potential access to employment, education, other meaningful activities, and including potential family involvement in treatment. The model also underlines the importance of working towards an inclusive community through people’s network and neighbourhood. Three FACT teams were included in the study covering one rural, one semi-rural and one urban area in Eastern parts of Norway. The study is part of a larger project involving the promotion of social inclusion among young adults in FACT teams.

### Recruitment and participants

FACT team staff were informed about the study and reached out to patients in the target group, providing them with information and a consent form to participate. All patients between the age of 18–30 were considered for recruitment. Some were excluded by staff due to high symptom levels at the time. The first author contacted and scheduled interviews with people who gave their consent to participate.

Seven participants (four women and three men) participated in the study, with ages ranging from 22–29 years (median = 27). All participants were born in Norway, and none had immigrant backgrounds. Three participants lived in rural regions, two in semi-rural regions and two in urban areas. Disclosed reasons for receiving services from FACT teams were mainly anxiety, depressive, psychotic, and neurodevelopmental disorders. Most participants reported having complex problems with more than one mental health disorder and limited everyday function. One participant disclosed present substance use and three past substance use, both illegal substances and alcohol. Three participants had histories of violence and two reported having extensive criminal histories. One was residing in a treatment facility, one was serving time in prison, and another participant had previously been imprisoned. Of the seven participants, one reported having a job, the rest had a history of failing to keep or obtain work. Four dropped out of school before or during high school. None had attained a degree in higher education, but two had tried and dropped out because of mental health and substance use problems. To ensure confidentiality and anonymity, all participants have been given pseudonyms.

### Data collection

Data was collected through individual semi-structured interviews from March to September 2023. This approach was chosen to enhance opportunities of rich data and in-depth understanding of participants’ subjective experiences through a flexible exploration (Denzin et al., 2023). Other reasons were the need to create a safe environment for a vulnerable target group, especially as regards potential discussion of emotionally-charged topics and experiences.

Four participants were interviewed in person at the FACT teams’ headquarters. Two participants were interviewed by telephone and one by online video interview. Two of the telephone and online interviews were participants preference over in-person interviews and one was due to the participant’s incarceration, with limited allowed visitation. Through a semi-structured interview guide developed by all authors, the first author conducted individual interviews with the aim of exploring participants’ experiences and views on social inclusion and ways to promote it for young adults such as themselves. In relation to this they were asked to describe who they were and how they perceived themselves and their perceived opportunities for social inclusion. They were also asked about the related concept of mattering, exploring their perceived self-value and how they added value. They were also asked to describe their views on people in their communities. Participants were then asked about their experiences of their FACT team’s role in promoting social inclusion. All were given the chance to contact the first author and provide additional statements after the interview, of which none did. As the interviews provided rich material with high levels of similar experiences, seven participants were considered sufficient. Length of interviews was 30 to 77 minutes, with an average duration of 56 minutes. Interviews were recorded and transcribed verbatim by the second and first authors.

### Analysis

The data was analysed following the six phases of reflexive thematic analysis (Braun & Clarke, 2021a) as discussed below. Using reflexive thematic analysis enabled us to give voice to the participants (Braun & Clarke, 2021b), which is crucial when studying identity and social inclusion. This approach ensures that the perspectives and experiences of individuals are central to the analysis, reflecting the social constructionist emphasis on the co-construction of meaning.

The first author led the process; however, a team approach was employed, with all contributing authors participating in the analysis. The first author and the peer researcher read and re-read the data and discussed initial impressions of the material, becoming familiarized with the dataset (phase 1). The peer researcher shared her initial impressions and thoughts first to minimize the possibility of the non-peer researcher’s influence. Initial impressions and reflections of the two authors had a high degree of similarity with no disagreements regarding each other’s impressions. The peer researcher also wrote summaries of the interviews, further enabling her familiarization with the material and intending to support her confidence in offering critical comments later in the process.

The first author proceeded with the second step of analysis, with coding of the material (phase 2), followed by discussions with all contributing authors. Following this, the first author proceeded with the generation of initial themes (phase 3), then developed and reviewed the themes (phase 4), and refined, defined, and named them (phase 5) in collaboration with other contributors. There were some movement back and forth between the different phases following discussions of the material before writing it up (phase 6).

The first author endeavoured to exercise critical awareness to avoid biases in data collection and interpretation of the data and through discussions with all authors during all phases of the analysis (Finlay, 2002). All contributors discussed the interview guide and re-read the material several times to re-examine initial findings. Two main themes were agreed upon (United Nations, 2016): “Who they are” with three subthemes: “A lifelong sense of being someone on the outside”, “Feeling different from others”, and “Someone who is worthless and doesn’t matter”, and (Filia et al., 2018) “Who they want to be”, with subthemes “Wanting to be someone else”, and “The need to redefine oneself”. See Table 1 for examples on how the analysis proceeded.

**Table 1.** Examples of analysis.

Statement	Initial coding	Sub-theme	Main theme
“I struggled academically in school, so when you combine that with not being popular, you’re just ostracized.”	Outsiderness Early experiences	A lifelong sense of being someone on the outside	Who they are
“I feel different and that I’m not able to get on with my life, like others on the same age.”	Feeling different	Feeling different from others	Who they are
“I don’t think I can do anything, it’s never right, I’m just not worth anything.”	Feeling worthless Not mattering	Someone who is worthless and doesn’t matter	Who they are
“I just wish I could contribute more. It could be anything. […] I don’t know if I’m able to do that.”	Wanting to contribute Hopelessness	Wanting to be someone else	Who they want to be
“I’m not a drug addict anymore. […] I can see that I am more [than that] now.”	Changing self-perception	The need to redefine oneself	Who they want to be

### Ethical considerations

The study was approved by the local data protection officer at Innlandet Hospital Trust (ID 22587532), following recommendations by the Regional Committee for Medical and Health Research Ethics in Norway (482463). The participants signed a consent form with information stating that the interviews were confidential that statements would have no impact on the services they received, and that they could withdraw from the study at any point. This information was repeated orally before the interviews started. Participants were told that their contributions and experiences were important, and they were given the chance to ask questions or voice concerns before participating in the study. None did. At the end of the interview, the researcher asked about the participant’s experience of the interview to ensure that no harm was inflicted. Participants received a gift card of 500 NOK for their contribution. Receiving a gift card for participation in research is not uncommon in Norway. Also, seen in relation to the study’s topic were contributing to society and being perceived as valuable is central, the gift card was a recognition of the participants contributions. There were no signs of this influencing the integrity of the research.

## Results

As above, reflexive thematic analysis resulted in two main themes. Theme one, “Who they are” has three subthemes: “A lifelong sense of being someone on the outside”, “Feeling different from others”, and “Someone who is worthless and doesn’t matter”. Theme two, “Who they want to be”, includes the subthemes “Wanting to be someone else”, and “The need to redefine oneself”. We used the pseudonyms Sophia, Mary, Jennifer, Catherine, Peter, James, and William for participants.

### Who they are

This theme highlights how the young adults reflected and talked about their identities during the interviews. Three subthemes are presented: “A lifelong sense of being someone on the outside”, “Feeling different from others”, and “Someone who is worthless and doesn’t matter”.

### A lifelong sense of being someone on the outside

The participants spoke of lifelong experiences of feeling excluded from society, with several stating that this sense had become a part of who they were. Participants talked about experiences in their own families and school as starting points for their feeling of outsider-ness. These experiences were described as influencing their sense of identity. All participants made statements such as “I’ve always felt like I’m on the outside” (Peter) and “it’s [sense of outsider-ness] just a part of me” (Jennifer).

For some participants, their experiences of exclusion started in their own families, through experiences of violence or not receiving the attention and affection they needed. James spoke of having had enough: “I simply feel excluded. When I get the chance to move out, I’m removing my father’s family name, I don’t want anything to do with them.” Catherine remembers the feeling of being on the outside stemming from observations of her friends and their responsible parents and comparing this with her own situation: “They [friend’s parents] would call during a movie night, but my parents never called me,” or from observing family dynamics during dinner at her friend’s house: “they [friends] could say something that would have made my parents explode, but their parents didn’t react, and I wondered why they didn’t. And I just concluded that, oh, it’s because they don’t do these things to their kids.” Coming under the care of the child protective services, with multiple moves between institutions and challenges with professional relationships, further increased people’s feeling of outsider-ness.

For other participants, a sense of social exclusion started in school with academic struggles or bullying. William, who was being bullied at school, started hanging out with an older neighbour who was using drugs and who offered him protection: “I just gradually started doing the things he did”, describing this as an important event leading up to the sense of being an outsider. Peter spoke of being beaten up daily by bullies and of feeling like an outsider from an early age. Jennifer shared similar experiences. She dropped out of school at age 13: “I used to enjoy going to school, I was kind of a nerd, but the bullying took over […], it made it difficult for me to show up”. This led to anxiety and isolation, further reinforcing her feeling of being excluded. James said: “If you had flown a drone over the school yard at winter, you would have seen a circle in the snow made by me, walking around and around the school building”, and continued “I struggled academically in school, so when you combine that with not being popular, you’re just ostracized.”

In total, participants described their experiences as fundamental in the development of their identities and as challenging their development as a member of society.

### Feeling different from others

The participants spoke of feeling different from others, both looking back and through comparing where they were in their lives at present. The feeling of differentness was often associated with not being employed or having a higher degree, not having started a family, having limited economic resources, or in comparison with picture-perfect lives on social media.

Sophia spoke of her sense of being different through being diagnosed with ADHD and said: “I feel different and that I’m not able to get on with my life, like others on the same age.” Jennifer talked about the constant reminder of being different and not fitting in when getting questions about what she does for a living and said: “If someone I don’t care about asks me that, I lie, I tell them I work somewhere I used to work before. […] I think it’s hard. Everyone has something to do, and I’ve got nothing.” Other participants also emphasized the effect of feeling different through unemployment or a history of failing to obtain or keep work, or not graduating from school. Jennifer connected the feeling of being different with previous experiences and social isolation: “After being bullied I shut myself in […] I never got the chance to act like other youths and do what they did”. James spoke of just feeling different from the start, having different interests than the other kids while growing up, and of having difficulties connecting with peers, preferring conversations with adults.

Mary also felt different from others. She talked about how she was affected by social media: “I just think it’s depressing seeing what other people are able to accomplish compared to me, getting a degree, buying a house, starting a family. I feel like I’m left behind in life.” All participants had a negative perception of social media, and several had deleted their accounts permanently or periodically. Catherine said this: “I’ve come to see that it’s no good for me, watching people’s perfect lives, and comparing myself with things that doesn’t matter, because it’s all a lie, really. […] I don’t’ miss it, it’s a relief.” Others had decided to keep their social media accounts despite feeling negatively about it in terms of differentness. Peter, declaring that he “hated’ social media, that it was “a waste of time,” and that it made him feel like a “braindead sheep”: “I deleted everything but Reddit where I can follow people like me […] I’ve got more in common with them compared to my old classmates. They’ve got their life together and I haven’t fucking accomplished anything.” He summed up his experiences with social media and differentness by saying “You just get so jealous, it makes me sick. Life is just not fucking fair, it should be more fairness in life.”

The sense of being different created insecurity and distance for participants, who wondered if they ever could fit in and be accepted as they were. The power of social media was evident in the interviews, as reinforcing people’s sense of differentness and creating a sense of hopelessness and injustice.

### Someone who is worthless and doesn’t matter

All participants talked about feeling worthless and not mattering, with this self-perception being connected to the sense of being different. Their statements related to this theme were not always specific, sometimes appearing to be a feeling and self-perception that was undisputed and integrated in their identity without their giving it much conscious thought. Examples related to this theme often revolved around experiences of growing up, work, education, and relationships. A sense of shame and internalized stigma was present in the material.

Jennifer stated: “At one point I got a dog, just to be important to someone” and then said that spending time with her family and friends or doing charity work helped somewhat, but that she still couldn’t stop feeling worthless and not mattering. James said: “I don’t think I can do anything, it’s never right, I’m just not worth anything”. Mary said the only times she felt she was worth something and mattered was when she could help someone, but she rarely had the chance to do so. Sophia said, “In many ways I feel useless. I feel like I’m not living, I just exist, if you know what I mean. I’m just here for some unknown reason, you know.” The same participant said that sharing about her mental health struggles om social media made her feel a little less worthless, as when she received feedback that her experience helped others, making them feel less lonely. Catherine had similar experiences related to practicing as a medium and connecting with people that had alternative ways of life, feeling worthless in the mainstream community.

### Who they want to be

This theme included two subthemes: “Wanting to be someone else”, and “The need to redefine oneself”. While this theme portrays people’s negative identities, it also reveals hope and wish to change.

### Wanting to be someone else

The theme derives from participants’ experience of being different, worthless and not mattering. All the participants spoke about wanting to be someone else. Most often, this involved the idea of being someone who contributed to society through working, connecting with others, and living a more social life. Participants also talked about wanting to be normal, with normal sometimes described as having your life in order in all domains of it.

Peter said: “I need to prove to myself, that I actually have something to contribute. […] It would be nice to see that I could actually do something [of value].” James said, “it would be nice to have a group of friends that could get together a couple of times a month […] and I would want a job with a good work environment where I could thrive, feel like I’m worth something, that I could perform and contribute to society”. Sophia had recent experiences of failing to keep her job because of her mental health and shared her disappointment; “I just wish I could contribute more. It could be anything. […] I don’t know if I’m able to do that.” James shared a similar experience after having to quit his job; “I just feel like I should’ve handled it,” he said, pointing to the importance of being a contributor even while trying to accept that working was not ideal for him at this point in his life.

Peter, speaking of the life he wanted, said, “I just don’t know if I’m able to have a normal life, that’s the thing. […] With a job, girlfriend, car, house, all of these things.” Jennifer also wanted a normal life, defining herself as abnormal, as she had tried to work several times and was currently receiving benefits. She later redefined normal to involve sufficient everyday function, partly related to a criticism of how a normal life is portrayed in social media.

### The need to redefine oneself

There were various degrees of consciousness among the participants about how their identities influenced their possibilities for social inclusion, their social position, and their views of society. Some participants said they hadn’t really thought about these things and didn’t know what to do about them, but “that sure feels like a lot of work,” said Peter. His sense of hopelessness became evident when he was asked to reflect on how he could redefine his identity to include a more optimistic self-perception: “I just feel like it’s how I’ll always be, really. […] I’m just used to it”. In general, when participants were asked about how their self-perception influenced their level of social inclusion, few had clear answers other than pointing to a general feeling of just not fitting in.

Some participants said that they didn’t know how to achieve greater social inclusion but were open to trying. “I need to believe it can change,” said James. William spoke of having to get his life together before he could even think about it [social inclusion]. Jennifer, who spoke of having spent her youth hiding or not finding out who she really was out of fear of being excluded or not being good enough, talked about wanting to address this issue after the delays and disruptions in her identity development; […] “I have to figure myself out. I was bullied, and I just wanted to be accepted. It was easier to just go along with what other people were saying, just agreeing with them”.

Some participants spoke of having started to redefine themselves. Catherine said “I’m not a drug addict anymore. […] I can see that I am more [than that] now”. Most participants pointed to the need of redefining and figuring this out for themselves, while some noted the importance of receiving support and being introduced to meaningful activities as a way of addressing their social exclusion. All in all, few thought the topic of identity and social inclusion was being addressed by service providers.

## Discussion

The aim and objective of this study was to explore the identities of young adults with mental illness and complex needs in relation to social inclusion, to provide practice-oriented knowledge on the subjective elements of and challenges to, promoting social inclusion. The stories that our participants told themselves about who they are and how they fit in confirm previous research showing that negative identity functions as a major barrier to social inclusion and that they experience various difficulties related to social inclusion (Corrigan & Watson, 2002; Gardner et al., 2019b; S. A. Kidd, 2003; S. Kidd & Davidson, 2007; Liljeholm & Bejerholm, 2020; Semb et al., 2019; Storm et al., 2023).

In addition, the results show the importance of combining objective and subjective perspectives when promoting social inclusion, with identity being one such important subjective and psychological factor. However, as previously noted, critiques suggest that the concept of social inclusion is ambiguous, raising the question on how social inclusion can be understood in terms of practical efforts to promote it. The services’ lack of ability to support people’s social lives may be affected by social inclusion as an intangible concept. This notion may be seen in relation to the results in this study showing that the young adults are doubtful as to how they can work to redefine their negative identities and whether this is possible, even with help from mental health and substance use services. In addition, participants reported that identity in relation to social inclusion was little addressed by the services. They also stated at the end of the interview that identity and social inclusion were important topics for them to talk about, even if they had limited consciousness of the topics initially.

The results also confirm the dynamic and interdependent relationship between personal, social, and societal factors in identity formation (Crocetti et al., 2018, 2023), embodying the complexity of both identity formation and promotion of social inclusion. The way the young adults discuss their identity reveals that it is strongly influenced by their social context and past and current life situation. Further, this seems to negatively affect their ongoing identity formation, which in turn has an impact on engagement and interaction with their social surroundings. This encompasses a negative circle leading to maintenance of their identity as a barrier for social inclusion.

We argue for the need to further develop existing frameworks of social inclusion with an equal emphasis between objective and subjective factors. Further, it seems necessary to include subjective psychological factors like identity in this work to enable service providers to effectively address these issues. Simply introducing and addressing identity and social inclusion as a relevant topic seems to be a good place to start. This may imply exploring how young adults perceive themselves in a social context and how they want to be perceived by themselves and others–examining what can be done to accomplish this, targeting obstacles and resources.

### Citizenship and mattering – and identity

The young adults in this study did not perceive themselves as valuable or able to add value to their surroundings, and emphasized the connection between identity, the ability and opportunities to contribute, and the experience of social inclusion. In addition, these young adults said they felt stuck in their identities and of having lifelong experiences that led to their sense of having negative identities. These findings imply the need for a systematic approach to subjective, as well as objective, factors with regard to promoting social inclusion through multifaceted means.

Our findings showed that our young adult participants had limited awareness of the concept of social inclusion, confirming previous research pointing at limited integration of social context in treatment (Johnson, 2017; Karadzhov, 2023; Norwegian Board of Health Supervision, 2019a, 2019b; The Office of the Auditor General, 2021; Topor et al., 2022; Ventevogel, 2014). Adoption of framework, such as citizenship, that aim to promote social inclusion, might clarify the concept and offer new strategies for young adults and service providers. Citizenship is defined by the 5 Rs of rights, responsibilities, roles, resources, and relationships that society offers its members through public and social institutions. It also emphasizes the sense of belonging in a society that must be validated by others (Rowe, 2015). Research shows the citizenship framework offers a promising approach for addressing social inclusion (Cogan et al., 2021; MacIntyre et al., 2019; Quinn et al., 2020). As identity was an important part of the framework in the beginning, citizenship progressed and largely left it behind as a focal point (Nord-Baade & Rowe, 2023). Citizenship might support new approaches to the development of positive identities, but people may have difficulties adjusting to new identities, hence returning to old ways of life (Rowe, 2015; Thulien et al., 2019). The self-perception of who one is, what one is worth, and how this affects where one belongs, might help to explain “relapse” to old ways and identities even in the context of improved life situations. Directly addressing these aspects might be of help to promote social inclusion.

As the results show that identity is connected to the notion of whether one is someone of value or have something of value to offer, employing the mattering framework might be a helpful way of addressing these subjective features of social inclusion. The young adults we interviewed did not feel as though they mattered. However, they expressed a strong need to contribute, and connected lack of social inclusion to their sense of worthlessness and perceived lack of ability to do so. The concept of mattering, which largely emphasizes subjective experiences through perceptions of self-value and adding value, has been proven to function as a facilitator to social inclusion (Prilleltensky & Prilleltensky, 2021; Prilleltensky et al., 2023). Mattering is ultimately a state of mind affecting both our identity and view on our position in society, with a strong connection to fairness, well-being, and hope (Flett, 2022; Paradisi et al., 2024), and a sense that you are a valuable and needed member of society.

The narrative identity approach also provides a valuable lens through which we can understand the impact of identity on social inclusion, as identities are formed and shaped by the stories we tell about ourselves, our experiences, and our place in the world (Arnett, 2000; McAdams, 2001; McLean & Syed, 2015). As our findings show, the narratives not only define how young adults perceive themselves but also influence how they interact with others and perceive their opportunities for social inclusion. The findings confirm previous research that shows how people with mental health and substance use problems face stigmatization and exclusion due to societal misconceptions and prejudices. These negative experiences shape self-narratives and create internalized stigma, often leading to a sense of isolation and a diminished sense of self-worth (Boyd et al., 2022). However, by understanding and applying the narrative identity approach, we can begin to address these issues, recognizing that narratives can change over time and allowing for the possibility of growth and transformation, even for people with life-long negative experiences dominating their identity.

Our suggestion is therefore to develop and combine the promising frameworks of citizenship and mattering with an emphasis on identity as a core facilitator of social inclusion. Adapting the narrative understanding of identity through exploring the stories they tell themselves on why and how they fit in or not, in combination with mattering and citizenship work, may be a possible approach to challenge an often rigid identity. This fusion of these frameworks might answer to the call for a multifaceted and tangible model to promote social inclusion, both for young adults with mental illness and complex needs and in promotion of social inclusion in general. As the concept of social inclusion may be intangible, developing such a model might enhance the services awareness and understanding of social inclusion as a multifaceted concept, and enhance their capability to promote it.

### Further research

Further research should investigate if the combination of mattering and citizenship with emphasis on identity may function as a more tangible approach to promote social inclusion in practice. This may be done through a conceptual development of the framework and intervention studies. Further, exploring the process of change over time in more subjective matters is needed due to lack of effective interventions, supporting the need for such research. In addition, further research should explore how service providers understand and integrate elements of social inclusion when working with young adults with mental illness and complex needs.

### Strengths and limitations

A team approach with different professionals was employed in the study, with the valuable combination of lived experience, psychological and sociological knowledge. The study could have benefitted from a higher number of participants. However, the data material was rich, and many participants had similar experiences and expressed similar sentiments, thus supporting the credibility of the results. In addition, all authors exercised critical awareness throughout the study. The participants lived in both rural, semi-rural and urban areas, suggesting that the results might be transferable to young adults in the target group independently of demographic context and in similar countries.

## Conclusion

The study points to the importance of addressing and handling the aspects of identity as it functions as a major barrier to social inclusion. The results also show limited awareness of social inclusion as a concept and a strong connection between social inclusion and the feeling of mattering among the young adults in the study. Combining and developing the frameworks of citizenship and mattering with an emphasis on a narrative identity approach to promote social inclusion is suggested. We call for both research and practical approaches.

### Declarations

Ethics approval and consent to participate: The study was approved the local data protection officer at Innlandet Hospital Trust (ID 22587532), following recommendations by the Regional Committee for Medical and Health Research Ethics in Norway (482463). All participants signed a written consent to participate after receiving information about the study.

Availability of data and materials: The data that support the findings of this study are available from the corresponding author, SNB, upon reasonable request.
